# PyEvoCell: an LLM-augmented single-cell trajectory analysis dashboard

**DOI:** 10.1093/bioinformatics/btaf158

**Published:** 2025-04-10

**Authors:** Sachin Mathur, Mathieu Beauvais, Arnau Giribet, Nicolas Aragon Barrero, Chaorui-Tom Zhang, Towsif Rahman, Seqian Wang, Jeremy Huang, Nima Nouri, Andre Kurlovs, Ziv Bar-Joseph, Peyman Passban

**Affiliations:** R&D Data and Computational Sciences, Sanofi, Cambridge, MA 02141, United States; R&D Data and Computational Sciences, Sanofi, Gentilly 94255, France; R&D Data and Computational Sciences, Sanofi, Barcelona 08019, Spain; R&D Data and Computational Sciences, Sanofi, Barcelona 08019, Spain; R&D Data and Computational Sciences, Sanofi, Toronto, ON M5V 1V6, Canada; R&D Data and Computational Sciences, Sanofi, Toronto, ON M5V 1V6, Canada; R&D Data and Computational Sciences, Sanofi, Toronto, ON M5V 1V6, Canada; Precision Medicine Computational Biology, Sanofi, Cambridge, MA 02141, United States; Precision Medicine Computational Biology, Sanofi, Cambridge, MA 02141, United States; Precision Medicine Computational Biology, Sanofi, Cambridge, MA 02141, United States; R&D Data and Computational Sciences, Sanofi, Cambridge, MA 02141, United States; R&D Data and Computational Sciences, Sanofi, Toronto, ON M5V 1V6, Canada

## Abstract

**Motivation:**

Several methods have been developed for trajectory inference in single-cell studies. However, identifying relevant lineages among several cell types and interpreting the results of downstream analysis remains a challenging task that requires deep understanding of various cell type transitions and progression patterns. Therefore, there is a need for methods that can aid researchers in the analysis and interpretation of such trajectories.

**Results:**

We developed PyEvoCell, a dashboard for trajectory interpretation and analysis that is augmented by large language model (LLM) capabilities. PyEvoCell applies the LLM to the outputs of trajectory inference methods such as Monocle3, to suggest biologically relevant lineages. Once a lineage is defined, users can conduct differential expression and functional analyses which are also interpreted by the LLM. Finally, any hypothesis or claim derived from the analysis can be validated using the veracity filter, a feature enabled by the LLM, to confirm or reject claims by providing relevant PubMed citations.

**Availability and implementation:**

The software is available at https://github.com/Sanofi-Public/PyEvoCell. It contains installation instructions, user manual, demo datasets, as well as license conditions. https://doi.org/10.5281/zenodo.15114803.

## 1 Introduction

Trajectory inference (TI) analysis is crucial to understanding cell differentiation and biological mechanisms (Ranek *et al.* 2022) in single-cell RNASeq experiments, particularly in development and dynamic biological processes (Pellin *et al.* 2019). While multiple TI methods exist (Saelens *et al.* 2019), very few tools assist users with interpreting results or identifying relevant lineages. A typical single-cell experiment consists of tens of thousands of cells and around 30 different cell types (also referred to as cell states in the literature) depending on the tissue and number of samples. Given the high dimensionality of data and sheer number of possible cell transitions (e.g. 2*30 choose 2 = 870), it is hard to identify relevant cell lineages in the context of the experiment even for well-trained biologists.

Dynverse (Saelens *et al.* 2019) allows users to compare multiple TI methods but does not help with lineage identification. Methods like Monocle (Trapnell *et al.* 2014) offer automatic node selection based on a cell type of interest, but users must specify the end node to define the trajectory. This limitation makes it difficult to explore cell transitions beyond domain expertise.

Following lineage identification, researchers typically perform downstream analysis such as differential gene expression (DGE), extract driver genes driving cell fate, and functional analysis using gene set enrichment analysis (GSEA) (Subramanian *et al.* 2005). While these provide useful information about the pathways involved, they are not always easy to interpret. Many genes are less known to researchers and GSEA often results in redundant list of several related pathways without providing a complete picture. A summary of relevant gene and biological mechanisms associated with the lineage would improve result interpretation and help users gain confidence in the results.

Large language models (LLMs) have shown promises in summarizing data in computational biology (Hao *et al.* 2024) but have not yet been applied to TI. To address these challenges, we developed PyEvoCell, a dashboard that integrates LLMs to enhance TI analyses. PyEvoCell helps with lineage identification and provides LLM-generated interpretations for lineages and their downstream analysis, such as extracting driver genes for cell fates, DGE and GSEA. We demonstrate the use of the PyEvoCell dashboard on Monocle3 trajectories for a KRAS inhibition dataset (Xue *et al.* 2020) and show its utility in identification of lineages, interpretation of downstream analyses, and how insights can be validated using PubMed references. It should be noted that PyEvoCell is designed to assist interpretation rather than compare LLMs or replace existing analysis tools.

## 2 Results

**Figure 1.**
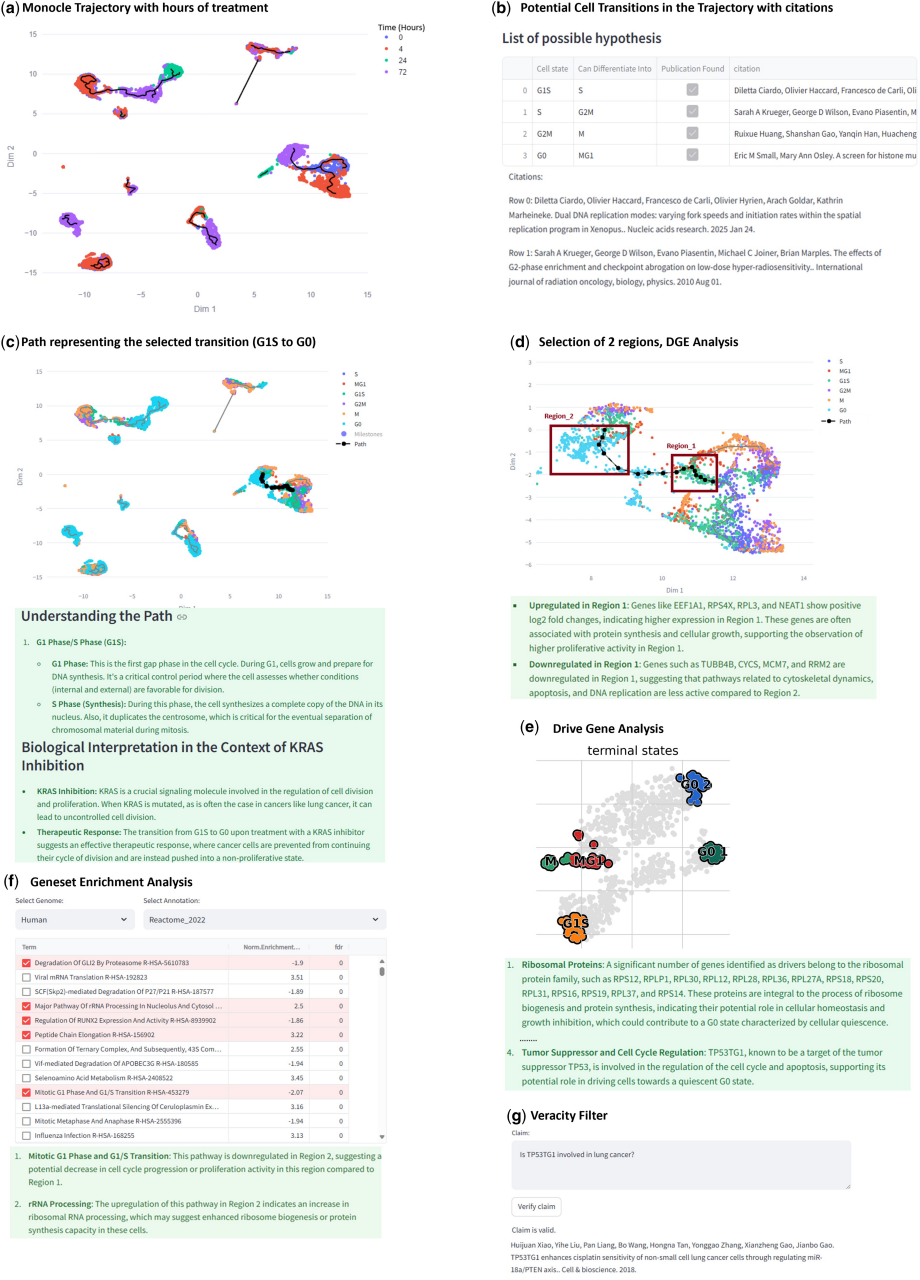
PyEvoCell features. (a) Monocle3 trajectory of the KRAS dataset that shows cells, color-coded by time of treatment (0, 4, 12, 72 h). (b) Recommendations of the Hypothesis Generation feature for cell transitions. (c) A path corresponding to the cell transition of interest (G1S to G0) highlighted in black with its explanation from the LLM. (d) Enlarged portion of the lineage (interactive feature offered in the dashboard) that contains the G1S to G0 transition and selection of two regions for DGE analysis, and interpretation of DGE results from LLM. (e) Terminal cell states calculated from CellRank along with explanation of results from the LLM. (f) Results of GSEA with Normalized Enrichment Score and FDR with a snippet of the interpretation from LLM. (g) Veracity Filter, where a claim can be queried to check its validity.

The KRAS dataset consists of 3 models of lung cancer that are treated with a KRAS inhibitor at 4, 24, and 72 h, in addition to untreated cells at 0 h (Xue *et al.* 2020) (details in supplementary file). We first visualize a Monocle3-generated trajectory within PyEvoCell for this data (Fig. 1a) where cells are color-coded by their treatment time. As seen, cells are often connected across time points indicating potential transitions of cells during the course of treatment. We next use the “Hypothesis Generation” option, a feature that uses cell types present in the trajectories to query the LLM to detect possible cell transitions. Plausible cell transitions along with their PubMed literature citation is displayed to the user (Fig. 1b) (It should be noted that EvoCell is not designed to be a comprehensive web-search tool for scientific discoveries, but it only tries to augment its claims with as much information as it finds from PubMed.). User-defined transitions (such as G1S to G0) can be manually added for exploration.

In this example, the transitions of cells in cell cycle are well known (Wang 2022) and each of them is supported by a publication. Nevertheless, the utility of this feature becomes particularly evident when applied to an extensive, complex, or lesser-known list of cell types. Since the cells are treated with a KRAS inhibitor whose goal is to stop cell proliferation, it would be of interest to see if paths exist along time that arrest cell cycle, such as G1S to G0, where G0 is the terminal state. As shown in Fig. 1c and d, a path from G1S to G0 that connects untreated cells (0 h) to cells 72 h following treatment is present. For this selected transition, an LLM-generated explanation indicates that the proliferation of cells observed in the initial part of the path is missing from the final phase of the path which can be the result of the KRAS inhibition treatment.

After selecting a path, the user can conduct differential expression analysis and/or extract driver genes. Figure 1a and c shows diverse cell types at different timepoints. Two regions; Region_1 and Region_2 (Fig. 1d) are selected using the interactive visualization tool and compared. Results are presented as a differentially expressed genes table providing information on log-fold change and adjusted *P*-value. The user can invoke the LLM feature to explain the genes that are different in the two regions and their activity in the context of KRAS inhibition (Fig. 1d). Though many differentially expressed genes are obtained from the DGE analysis, the LLM highlights the upregulation of specific genes such as EEF1A1, RPS4X, NEAT1 in protein synthesis and cellular growth as the main differentiators between the two regions and postulates that they are likely involved in the shift toward G0 in Region_2. Additionally, driver genes leading to the terminal cell state G0 can be extracted. Figure 1e shows the possible terminal states in the path selected by the user and the LLM explanation highlights the role of ribosomal genes in driving cells to the G0, while TP53TG1 is shown to be a cell cycle regulator.

Next, GSEA (Subramanian *et al.* 2005) can be performed in PyEvoCell on the DGE list by choosing the appropriate genome and annotation of interest. A table of biological mechanisms/pathways with normalized enrichment scores and FDR values is presented. The user can select mechanisms of interest and obtain an explanation of how they relate to each other in the context of the comparison (Fig. 1f). In this example, for the five statistically significant pathways from Reactome (Vastrik *et al.* 2007), the LLM explanation reiterates finding from driver genes on ribosomal genes and dysregulation of cell cycle following treatment with the KRAS inhibitor.

Finally, the association of TP53TG1 with lung cancer, an observation from the driver gene analysis, can be checked via the “veracity filter” (Fig. 1g), where it shows that an association exists and retrieves the appropriate publication. For more details about the entire process including LLM prompts, see the supplementary file. The results from an additional dataset (Pancreas) in PyEvoCell are present in the supplementary file.

### 2.1 Technical details

PyEvoCell is developed in Python 3.10 and relies on Streamlit (https://streamlit.io/) as its UI component, making it an interactive application. In the trajectory, each milestone is associated with the most prevalent cell state. Connected regions in the trajectory with milestones belonging to the user-defined start and end cell states are identified, and paths of various lengths are constructed. The user can then specify the “number of neighbors” parameter to visualize the path among cell states.

The Driver Gene component uses the CellRank 2.0 (Weiler *et al.* 2024) and relies on the pseudotime from the starting state specified by the user. The DGE and GSEA analyses are enabled by PyDeSeq2 (Muzellec *et al.* 2023) and GSEApy (Fang *et al.* 2023), respectively. For LLM capabilities, users can use open-source alternatives through Ollama (https://github.com/ollama/ollama). GPT models are also supported within PyEvoCell. Obviously, for GPT models, an API key will be required that can be set in the code (see installation instructions).

The input to PyEvoCell is a set of files that consists of metadata, a count matrix, and the trajectory itself that has been converted from a Monocle3 CDS R object to CSV. This conversion can be achieved by users’ in-house solutions or publicly available scripts such as those listed in the manual. The Github repository consists of a user manual that details data formats and step-by-step instructions, installation instructions, and demo datasets.

### 2.2 LLM considerations

To minimize hallucinations, we use data-specific details such as cell types, milestones, experiment context to control the LLMs output. Instead of broad scientific explanations, we guide the output generation by providing highly detailed prompts and context, ensuring it focuses on the precise phenomenon of interest. Specifically, during hypothesis generation, after prompting the LLM to extract potential cell state transitions from the list of all cell states present in the dataset, the LLM is asked to retrieve at least three PubMed publication titles for each plausible state transition. The publication titles are checked against the PubMed database by using partial word match with at least five consecutive word matches after accounting for word variants through the PubMed API call. These filtered results along with the plausible cell state transitions are displayed to the user.

We term the prompt with the extra steps to verify publications related to cell state transitions as the “engineered prompt.” To evaluate reproducibility and accuracy of results from the LLM, we compared the engineered prompt to a non-sophisticated (naïve) prompt that only checks for possible cell state transitions (details in supplementary file). Supplementary Table S1 in the supplementary file under section “Evaluation of LLM Prompts” shows the performance metrics of reproducibility and effect of different prompting strategies at various temperatures.

The precision of the engineered prompt is consistently higher than the naïve prompt, although recall is low. The higher standard deviation in the engineered prompt is because of cross-checking of publications, whereas the naïve prompt consistently retrieves the same information. In our preferred setup (temperature = 0.7), we achieve a considerably high precision, indicating that only the most confident results are presented. The low recall is intentional and by design. A potential shortcoming with this approach is that it may not identify all the transitions or verify every claim; however, despite these limitations, we believe LLMs add significant value, particularly for complex datasets in explaining trajectories, and downstream results. We hope this work serves as a starting point for the scientific community to explore this direction with more refined and controlled approaches.

## 3 Conclusion

PyEvoCell addresses an unmet need for analyzing trajectories in single-cell RNA-Seq datasets. Leveraging a large body of knowledge through an LLM, it facilitates the identification of cell transitions and paths of interest within trajectories, significantly saving researchers time and effort. Additionally, it supports downstream analysis of cell lineages by providing insights into key drivers of cell fates, DGE, and GSEA results in the context of the experiment. We demonstrate that customized prompting leads to improved outcomes, suggesting a promising future direction for research in this area.

## Supplementary Material

btaf158_Supplementary_Data

## References

[btaf158-B1] Fang Z , Liu X, Peltz G. GSEApy: a comprehensive package for performing gene set enrichment analysis in Python. Bioinformatics 2023;39:btac757. 10.1093/bioinformatics/btac757 PMC9805564 36426870

[btaf158-B2] Hao M , Gong J, Zeng X et al Large-scale foundation model on single-cell transcriptomics. Nat Methods 2024;21:1481–91. 38844628 10.1038/s41592-024-02305-7

[btaf158-B3] Muzellec B , Teleńczuk M, Cabeli V et al PyDESeq2: a python package for bulk RNA-seq differential expression analysis. Bioinformatics 2023;39:btad547. 10.1093/bioinformatics/btad547 PMC10502239 37669147

[btaf158-B4] Pellin D , Loperfido M, Baricordi C et al A comprehensive single cell transcriptional landscape of human hematopoietic progenitors. Nat Commun 2019;10:2395. 31160568 10.1038/s41467-019-10291-0 PMC6546699

[btaf158-B5] Ranek JS , Stanley N, Purvis JE. Integrating temporal single-cell gene expression modalities for trajectory inference and disease prediction. Genome Biol 2022;23:186. 36064614 10.1186/s13059-022-02749-0 PMC9442962

[btaf158-B6] Saelens W , Cannoodt R, Todorov H et al A comparison of single-cell trajectory inference methods. Nat Biotechnol 2019;37:547–54. 30936559 10.1038/s41587-019-0071-9

[btaf158-B7] Subramanian A , Tamayo P, Mootha VK et al Gene set enrichment analysis: a knowledge-based approach for interpreting genome-wide expression profiles. Proc Natl Acad Sci USA 2005;102:15545–50. 16199517 10.1073/pnas.0506580102 PMC1239896

[btaf158-B8] Trapnell C , Cacchiarelli D, Grimsby J et al The dynamics and regulators of cell fate decisions are revealed by pseudotemporal ordering of single cells. Nat Biotechnol 2014;32:381–6. 24658644 10.1038/nbt.2859 PMC4122333

[btaf158-B9] Vastrik I , D'Eustachio P, Schmidt E et al Reactome: a knowledge base of biologic pathways and processes. Genome Biol 2007;8:R39. 17367534 10.1186/gb-2007-8-3-r39 PMC1868929

[btaf158-B10] Wang Z. Cell cycle progression and synchronization: an overview. Methods Mol Biol 2022;2579:3–23. 36045194 10.1007/978-1-0716-2736-5_1

[btaf158-B11] Weiler P , Lange M, Klein M et al CellRank 2: unified fate mapping in multiview single-cell data. Nat Methods 2024;21:1196–205. 38871986 10.1038/s41592-024-02303-9 PMC11239496

[btaf158-B12] Xue JY , Zhao Y, Aronowitz J et al Rapid non-uniform adaptation to conformation-specific KRAS(G12C) inhibition. Nature 2020;577:421–5. 31915379 10.1038/s41586-019-1884-x PMC7308074

