# Supplementary material for: PyEvoCell: an LLM-augmented single-cell trajectory analysis dashboard
Source: Bioinformatics. 2025 Apr 10;41(4):btaf158. doi: 10.1093/bioinformatics/btaf158 (PMC12014098; doi:10.1093/bioinformatics/btaf158)
Supplement: btaf158_Supplementary_Data [file btaf158_supplementary_data.doc]

# Supplementary Information

# Datasets

## KRAS (discussed in the paper)

KRAS G12C-mutant tumor cell models (H358, H2122 and SW1573) were treated with the KRAS G12C (ARS1620, 10µM) inhibitor for 0, 4, 24 and 72h, followed by rapid collection of attached cells. The count data was downloaded from <https://www.ncbi.nlm.nih.gov/geo/query/acc.cgi?acc=GSE137912> . The initial processing of the dataset was performed using “import_KRAS.R”.[[1]](#footnote-2) The dataset consists of ~10k cells. The cell cycle process starts with G1 where cell organelles are replicated, and the cell then transitions to S where DNA is replicated. G2 serves as a checkpoint before the cell is split into two during mitosis. G0 is the quiescent state.

## Pancreas Dataset (discussed in this manuscript)

The pancreas dataset was generated from 4 embryonic stages (E12.5-15.5) of pancreatic epithelial cells from Neurogenin3 (Ngn3)-Venus fusion (NVF) homozygous mice. Endocrine progenitor cells (NVF+) were enriched by FACS cell sorting. The RNASeq gene count data is available at <https://www.ncbi.nlm.nih.gov/geo/query/acc.cgi?acc=GSE132188>. Samples at timepoints “12.5” and “15.5” were selected for our experiment.  The dataset consists of ~21k cells.

# Input Data Requirements

The data and the application must be in the same directory. Details of the files consumed by the application as its input are as follows: .

- Metadata: CSV file that contains column cell_id for the cell identifier
- Monocle trajectory: The CDS file must be converted to a CSV format (by the script at <https://github.com/mbeauvai/monocle3-cds2csv> or any similar script). The script produces 5 output files, including:
  - progressions.csv,
  - milestone_percentages.csv,
  - dimred_milestone.csv,
  - dimred.csv,
  - and trajectory_edges.csv
- Count Data: A comma delimited count data file (count_data.csv) that has gene names in rows and cell ids as column names.

The cell identifiers of the count_data.csv and metadata must be identical.

# LLM Features

The LLM is integrated in the application with hypothesis generation, DGE, GSEA, and veracity filter. It should be noted that our prompts are defined in the application and some parts of them are generated dynamically, meaning users only click on the LLM features and see the final output and all the details (prompt engineering, pulling information from different modules etc) are hidden from them. Thus, users do not need to deal with the complexities of prompt engineering or providing the right information within the prompt.

In our setup, each prompt is divided into three key components: context, static, and dynamic. The context component captures the user-provided information related to the experiment, such as details about a specific disease or tissue dataset. This information is automatically incorporated into the prompt. The static component contains the core message or request we aim to communicate to the LLM. For example, in the case of Hypothesis Generation, the main request might be to generate a list of possible cell transitions. Finally, the dynamic component includes supplementary, yet essential information that enhances the LLM's accuracy. For instance, a list cell types, automatically retrieved from other modules of the application, is added to the prompt without any user intervention. Example of prompts used in the application are listed below with static text enclosed in [static] …. [/static] and dynamically generated text enclosed in [dynamic] …. [/dynamic]. These additional symbols are provided for clarity to help the reader better understand our prompts and are not part of the original prompts.

If it is a time series experiment, the prompt is changed such that it also asks for changes with respect to time. See examples for more detail.

## LLM Prompts

### Hypothesis Generation

Input: Trajectory from Monocle3 for the KRAS dataset

Prompt:

[static] You are expert in the single cell RNASeq domain, especially in understanding cell state transitions. [/static]

[context] The context of the dataset is within parenthesis (Dataset consists of lung cancer cells that have been treated with a KRAS inhibitor at 4, 24 and 24 hours. Cells were untreated at 0 hour.). [/context]

[dynamic] You are an expert in understanding cell transitions. Given the list of cell types provided here, please list the transitions among the following cell type. Send the output as a string in the following format: Initial Cell State:Transition;Initial Cell State:Transition;... Only the string, no other comment or explanation. The list of celltypes are: S, MG1, G1S, G2M, M, G0 [/dynamic]

Once the output is received then the publication is retrieved that support the transition.

[dynamic] Checking transition 1/17 from ' G1S ' to ' G2M ' with LLM and pubmed

Do G1S cells transition to G2M cells? Do G1S cells differentiate to G2M cells? Please give response as yes or no only. If yes, then retrieve 3 complete titles of articles from pubmed and make sure the paper titles exist. Output the results in the exact format here:

Yes/No

Title1:

Title2:

Title3:

[/dynamic]

### Path/Lineage Explanation

Input: A path that consists of milestones that is provided as part of the output in the trajectory inference method.

In turn, a milestone can comprise of multiple cell types and the most frequent cell type is assigned to a given milestone. The progression of the path/lineage along the milestones in the context of cell types is given to the LLM as text. In addition, the context of the experiment is also provided to the LLM.

[context] The context of the dataset is within parenthesis (Dataset consists of lung cancer cells that have been treated with a KRAS inhibitor at 4, 24 and 24 hours. Cells were untreated at 0 hour.). [/context]

<static> Given the cell differentiation path below, tell me about the path and talk about cell type evolutions. For your information what you see in the path is a sequence of milestones and the path is created by Monocle 3 or a similar tool. Try to focus on explaining the path from a biology perspective. </static>

<dynamic> The path is: G2M, G2M, G2M, G2M, G2M, G2M, M, G2M, M, M, M </dynamic>

EXAMPLE

### DGE Analysis

Inputs: A DGE table obtained from pyDeSeq2, context of the experiment provided by the user, and cell type distribution in the 2 regions

Prompt:

[static] You are expert in single cell rnaseq analysis.[/static]

[context] The context of the dataset is within parenthesis (Dataset consists of lung cancer models treated with a KRAS inhibitor at 0, 4, 24 and 72 hours). [/context]

[dynamic] We are comparing 2 regions. Region 1 has celltypes with their proportions listed within parenthesis (G1S is 20.15%, S is 5.83%, and MG1 is 1.21%.). Region 2 has celltypes with their proportions listed within parenthesis (S is 44.66%, G2M is 11.17%, G1S is 9.22%, M is 6.31%, and G0 is 1.21%. ).The top 25 differentially expressed genes between region 1 and region 2 are listed within parenthesis (HNRNPA2B1, PSMA4, ENO1, ANXA2, RPS12, CTGF, LDHA, RTN4, RPLP1, KRT18, PKM, RAN, PCNA, PA2G4, RPS18, CCT6A, VDAC1, CCT5, CYR61, ARF4, CCT2, RPL41, SRSF7, PPIB, ATP5B). This is a time series experiment and cells in the 2 regions have different distributions. In Region_1, 69.86% of cell are at time 0, 19.3% are at time 4. In Region_2, 89.67% of cells are at time 72. Please comment if there was any difference in the regions with respect to time. [/dynamic]

[static] what are the main differences between these two regions from a biology perspective? Please focus on the biological differences between the 2 regions. Only list genes that explain the difference between the 2 region. [/static]

### Drive Genes

Inputs:

- The path chosen by the user – cells included in the path
- Starting cell state specified by the user
- End cell state specified by the user

Prompt:

[static] You are expert in single cell rnaseq analysis.[/static]

[context] The context of the dataset is within parenthesis (Dataset consists of lung cancer models treated with a KRAS inhibitor at 0, 4, 24 and 72 hours). [/context]

[dynamic] The objective is to find the driver genes for the cell fates. The terminal cell state is listed within parenthesis (< terminal_celltype>). The top 25 genes identified as drivers are listed within parenthesis ( <genes>). List genes that are responsible for cells to progress towards the terminal cell state <terminal_celltype>. [/dynamic]

[static] Please summarize the conclusions that can be drawn from this. Limit the explanation to only genes that were found to be important. [/static]

### GSEA

Inputs: Results obtained from PyGSEA, selected biological mechanisms by the user, context of the experiment

Prompt:

[static] You are expert in single cell rnaseq analysis. [/static]

[context] The context of the dataset is within parenthesis (Dataset consists of lung cancer models treated with a KRAS inhibitor at 0, 4, 24 and 24 hours.) [/context]

[dynamic] We are comparing 2 regions. Region 1 consists of the celltypes listed within parenthesis (G1S, S, MG1). Region 2 consists of celltypes listed within parenthesis (S, G2M, G1S, M, G0). Geneset enrichment analysis between Region_1 and Region_2 show that, Hh Mutants Aborgate Ligand Secretion is down regulated in Region 2, S Phase is down regulated in region 2, CDK-mediated Phosphorylation And Removal Of Cdc6 is down regulated in region 2. [/dynamic]

[static]  Please summarize the conclusions that can be drawn from this in the context of the comparison? Please skip the explanation of pathways. [/static]

### Veracity Filter

In our approach, when a claim is made, it is first evaluated using the LLM along with the relevant experimental context to determine its validity. If the LLM confirms the claim as valid, it then retrieves up to three relevant publications. These publications are subsequently searched in PubMed using its API.

The process of finding a paper begins by querying the LLM for the exact title. Given the significant advancements in LLM capabilities, they can often provide highly relevant titles. If the exact title/paper exists in PubMed, we show it directly. If not, we extract n-grams (n consecutive words) from the title suggested by the LLM. These n-grams, serve as a proxy to locate similar papers. A publication containing those n-grams in its title could be a suitable candidate for further review.

It is important to note that this module is not a fully-fledged semantic search engine. Rather, we have integrated this straightforward search mechanism into our PyEvocell platform to assist users in finding relevant publications efficiently and querying PubMed directly within the platform.

# Evaluation of LLM Prompts

To minimize hallucinations, we use data-specific details such as cell types, milestones, experiment context to control the LLMs output. Specifically, during hypothesis generation, after prompting the LLM to extract potential cellstate transitions from the list of all cellstates present in the dataset, the LLM is asked to retrieve at least 3 PubMed publication titles for each plausible cellstate transition. The publication titles are checked against the PubMed database by using partial word match with at least 5 consecutive word matches after accounting for word variants through the PubMed API call.

We term the prompt with the extra steps to verify publications related to cellstate transitions as the “engineered prompt”. To evaluate reproducibility and accuracy of results from the LLM, we compared the engineered prompt to a non-sophisticated (naïve) prompt that only checks for possible cellstate transitions.

There were 6 cell states in the KRAS dataset - G0, G1S, G2, G2M, M, MG1.

For Hypothesis Generation, the naïve prompt was as follows:

“*You are expert in the single cell RNASeq domain, especially in understanding cell state transitions. Given the list of cell types provided here, please list the transitions among the following cell type. Send the output as a string in the following format: Initial Cell State:Transition. Only the string with no other comment or explanation. Please separate the entries with 3 spaces and number them.
The list of celltypes are: S, MG1, G1S, G2M, M, G0”*

The engineered prompt is already described in the previous section – “Hypothesis Generation”.

We established a benchmark by listing all the possible cellstate transitions among the 6 cellstates using review articles from PubMed. The True Positive set included 7 cell state transitions; G0->G1S, G0->S, M->G1S, G1S- >S, S->G2M, G2M->M, M->G1S. These 7 transitions were used to evaluate the results of the 2 prompts – Engineered Prompt and Naïve Prompt. The citations obtained from the engineered prompt were cross checked for veracity. OpenAI GPT-4o was used for retrieving the results from both prompts.

Table S1 lists the performance metrics of the reproducibility and accuracy of the engineered and naïve prompts.

|  | Engineered Prompt | | | Naive Prompt | | |
| --- | --- | --- | --- | --- | --- | --- |
| Temperature | recall | precision | f-score | recall | precision | f-score |
| 0.1 | 0.28(0.08) | 0.87(0.17) | 0.41 (0.09) | 0.67(0.0) | 0.67(0.0) | 0.67 (0.0) |
| 0.3 | 0.33(0.14) | 0.83(0.24) | 0.46 (0.15) | 0.67(0.0) | 0.67(0.0) | 0.67 (0.0) |
| 0.5 | 0.27(0.12) | 0.77(0.32) | 0.38 (0.16) | 0.62(0.08) | 0.64(0.06) | 0.63 (0.07) |
| **0.7** | **0.48(0.21)** | **0.98(0.06** | **0.62 (0.21)** | 0.6(0.09) | 0.63(0.06) | 0.61 (0.07) |
| 0.9 | 0.27(0.12) | 0.76(0.22) | 0.38 (0.13) | 0.57(0.09) | 0.64(0.06) | 0.6 (0.07) |

Table S1: Performance metrics for engineered prompt and a prompt without publication verification (naïve prompt) at different temperatures for openai-4o model. The mean of performance metrics is listed (10 repetitions) along with the standard deviation within parenthesis.

To account for the inherent variability in LLM outputs, each prompt was submitted to the LLM 10 times, with a 10-second pause between each submission to minimize potential dependencies between responses. This iterative process was carried out across a spectrum of temperature settings (0.1, 0.3, 0.5, 0.7, 0.9), which control the randomness of the LLMs output. For each temperature, the mean and standard deviation of the performance metrics (recall, precision, f1-score) were calculated for both the engineered and naïve prompts. This allowed for a quantitative comparison of prompt performance at different levels of LLMs creativity.

The prompts to the LLM were repeated 10 times with 10 seconds gap at each temperature. The mean and standard deviation were calculated at each temperature for both the engineered and naïve prompts.

# Findings From Pancreas Dataset

We used the pancreas dataset in PyEvoCell (in addition to the dataset explored in the paper) to provide more results from our application and ensure the reader that it can generalize to their datasets too. Unlike the KRAS dataset, the pancreas dataset has cells differentiating into different celltypes.

Results are summarized in Figure S1. S1a shows the Monocle trajectory. Hypothesis generation from LLM points to many known cell type transitions in Figure S1b. Given Ngn3_High_late celltype is involved in differentiation of beta, alpha and other cell types (Soyer, Flasse et al. 2010), we chose to explore the transition of Ngn3_High_late to beta cells. S1c shows the path of differentiation from Ngn3_High_late to beta cells. We pick two regions as shown in S1d corresponding to the aforementioned cell types, and compare them using DGE. Results in S1e indicate genes Pyy (Khan, Vasu et al. 2016), Iapp, Ins2 are some of the prominent markers of mature endocrine cell types such as beta cells. The time component is also related to the differentiation of cell with the differentiation being complete at day 15.5, while undifferentiated Ngn3_High_late are prominently in 12.5 days. Role of Neuro3 is also highlighted in the LLM explanation.

We next perform GSEA on the DGE results and obtain explanation from the LLM as shown in S1f. It lists cell proliferation and metabolic activity is down regulated and explains that this may be due to the fact that cells in Region 2 are much more differentiated compared to Region 1 that comprised of mainly the earlier timepoint. Further the claim that Pyy gene is involved in beta cell differentiation can be verified through the veracity filter, as shown in S1g.


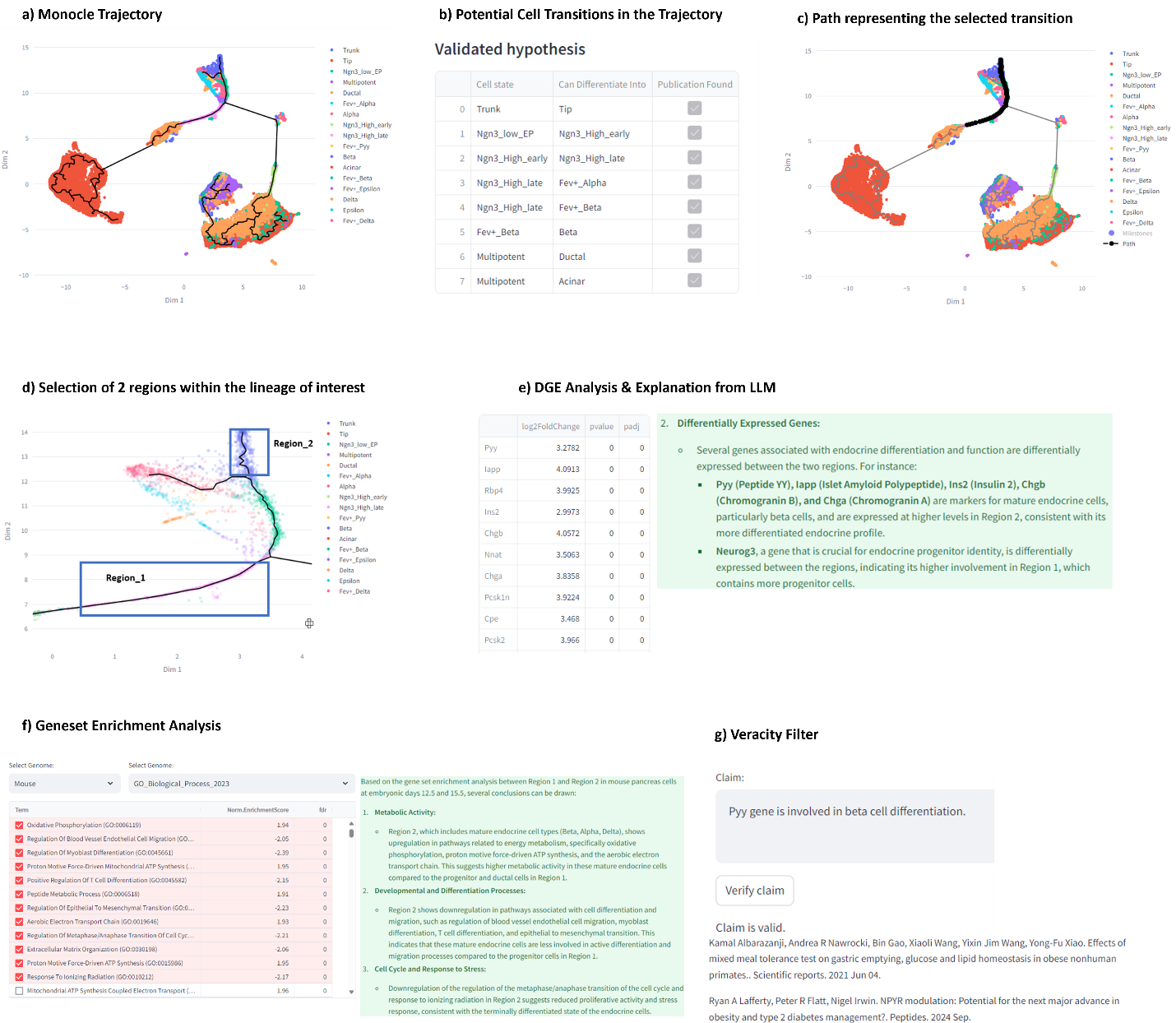


Figure S1a) Monocle3 trajectory of the Pancreas dataset that shows cells, color-coded by cell types. S1b) Recommendations of the Hypothesis Generation feature for cell transitions. S1c) A path corresponding to the cell transition of interest (Ngn3_High_late to beta cells) highlighted in black with its explanation from the LLM. S1d) Enlarged portion of the lineage (interactive feature offered in the dashboard) that contains the Ngn3_High_late to beta cells transition and selection of two regions for DGE analysis. S1e) Results of DGE analysis accompanied with interpretations generated by the LLM. S1f) Results of GSEA with Normalized Enrichment Score and FDR with a snippet of the interpretation from LLM. S1g) Results from the Veracity Filter where the user puts a claim. is missing

# References

Khan, D., S. Vasu, R. C. Moffett, N. Irwin and P. R. Flatt (2016). "Islet distribution of Peptide YY and its regulatory role in primary mouse islets and immortalised rodent and human beta-cell function and survival." Mol Cell Endocrinol **436**: 102-113.

Soyer, J., L. Flasse, W. Raffelsberger, A. Beucher, C. Orvain, B. Peers, P. Ravassard, J. Vermot, M. L. Voz, G. Mellitzer and G. Gradwohl (2010). "Rfx6 is an Ngn3-dependent winged helix transcription factor required for pancreatic islet cell development." Development **137**(2): 203-212.

1. <https://github.com/HectorRDB/bioc2021trajectories/blob/main/R/import_KRAS.R> [↑](#footnote-ref-2)
